# Innominate artery bifurcation pseudoaneurysm repair by “kissing stent-grafts technique”: a case report

**DOI:** 10.1186/s13256-018-1840-7

**Published:** 2018-11-27

**Authors:** Xin Li, Chang Shu, Quan Ming Li, Kun Fang, Ming Li, Wenwu Cai

**Affiliations:** 1 Vascular Surgery Department, Second Xiangya Hospital, Central South University, No. 139 Renmin Road, Chang Sha, Hunan, Changsha, 410011 People’s Republic of China; 2 State Key Laboratory of Cardiovascular Disease, Center of Vascular Surgery, Fuwai Hospital, National Center for Cardiovascular Disease, Chinese Academy of Medical Sciences and Peking Union Medical College, Beijing, 10037 People’s Republic of China; 3 Vascular Disease Institute, Central South University, No. 139 Renmin Road, Chang Sha, Hunan Changsha, 410011 People’s Republic of China

**Keywords:** Innominate artery, Pseudoaneurysm, Kissing stent grafts

## Abstract

**Background:**

We introduce the “kissing stent-grafts technique” for a patient who suffered from a pseudoaneurysm in bifurcation of innominate artery. This technique repaired an innominate artery bifurcation pseudoaneurysm; it successfully isolated the pseudoaneurysm and preserved both right subclavian and right common carotid artery.

**Case presentation:**

A 60-year-old Asian (Chinese) woman complained of discovering a cervical pulsatile mass. A pseudoaneurysm at the location of innominate artery bifurcation is a rare and difficult situation that should be treated by vascular surgeons. To our knowledge, this is the first case to use the “kissing stent-grafts technique” in treating innominate bifurcation pseudoaneurysm. With this minimally invasive endovascular treatment, our patient avoided open surgery and recovered quickly.

**Conclusions:**

When treating vascular lesions with complicated anatomy, endovascular treatment always has the merit of being minimally invasive. “Kissing stent-grafts technique” can be useful in locations other than coronary and aortic bifurcation.

## Background

Pseudoaneurysm at the location of innominate artery bifurcation is a rare and difficult situation that should be treated by vascular surgeons. A review of the literature showed that reports of a pseudoaneurysm at this location are very rare. Only two studies reported a lesion causing stenosis and obstruction at the innominate artery bifurcation. The “kissing stent-grafts technique” comes from the kissing stents technique, which is a smart manipulation to treat a lesion in vessel bifurcation usually in coronary artery angioplasty and stenting. However, this technique is not always used in bifurcation lesions other than coronary artery and aortic bifurcations. This is the first case report of the use of an endovascular method to repair an innominate artery bifurcation pseudoaneurysm.

The “kissing stent-grafts technique” used in this special anatomic location to treat the pseudoaneurysm is a kind of unusual creativity. In an endovascular era, there is still some “hostile” vessel anatomy which limits invasive treatment. With current devices, using kissing stent grafts in this location is creative in some ways. It solved the problem of large different diameter of two arteries and blood leak into the pseudoaneurysm.

## Case presentation

### Patient information

A 60-year-old Asian (Chinese) woman complained of discovering a cervical pulsatile mass. She also complained of dysphagia and dyspnea symptoms. She has a history of Meniere’s disease.

### Clinical findings

When admitted in the vascular surgery ward, her vital signs were stable and she complained of dizziness. A solid pulsatile 4 × 4 × 5 cm mass could be palpitated at the right cervical and supraclavicular fossae. There was no tenderness of the mass. The pulse of her right carotid artery and right branchial artery were normal. Her body temperature was 36.7 °C, heart rate was 108/minute, respiration rate was 20/minute, and oxygen saturation was 98% at administration. Bilateral upper extremity blood pressure was equal at 132/70 mmHg. The laboratory findings were white blood cells (WBC) 10.01 × 10^9^/L and neutrophils 6.92 × 10^9^/L; a liver function test revealed: aspartate aminotransferase (AST) 10.8 μmol/L, albumin 39.7 g/L, and D-dimer 0.92 μg/mL. There was no special family history or other genetic information of our patient. She has no history of smoking tobacco and alcohol consumption. There was no special prior social, employment, and environmental history of this patient.

### Diagnostic assessment

**Fig. 1:**
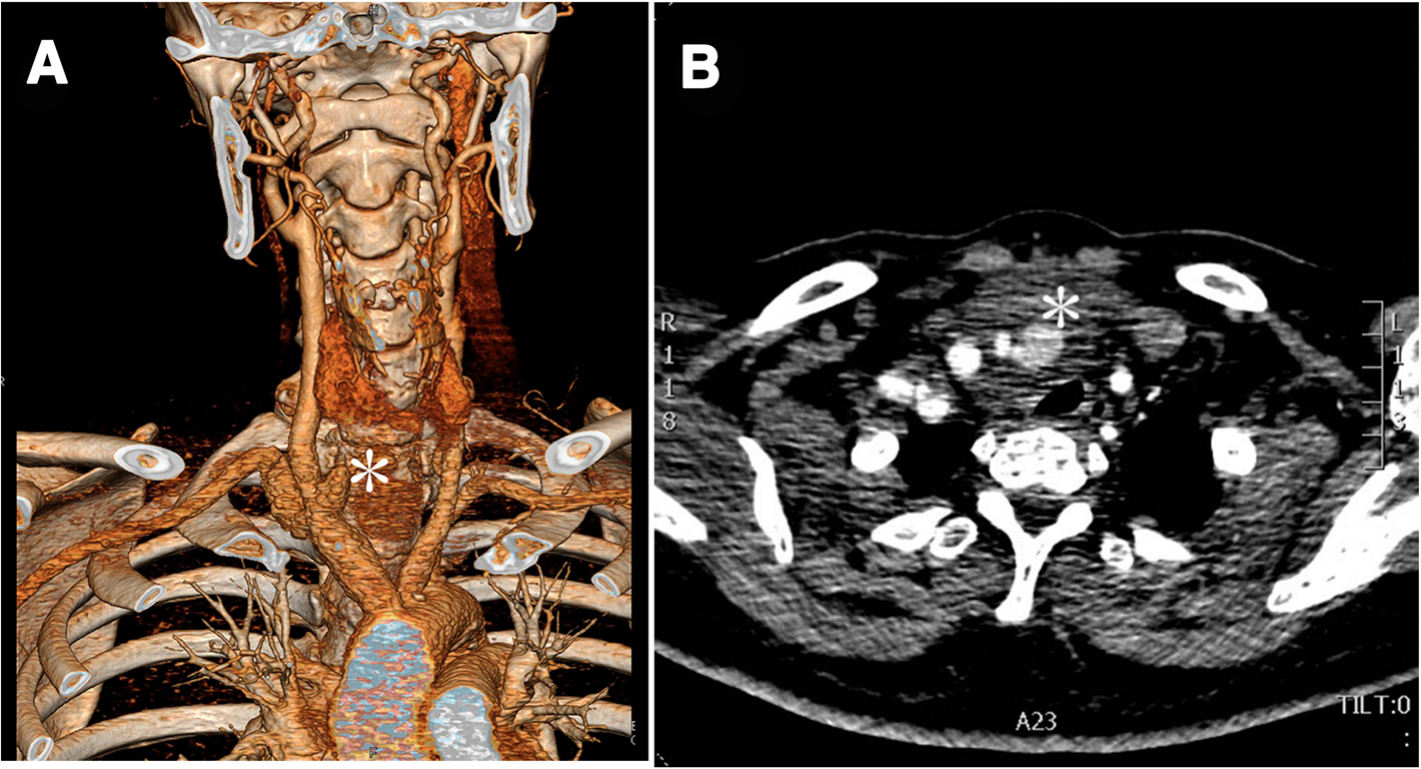
**a** Computed tomography angiography shows that the rupture area is located on the bifurcation of the innominate artery. *Star mark* (*) shows the position of the pseudoaneurysm. **b** Transverse computed tomography slide shows the pseudoaneurysm (white star mark *); trachea is compressed slightly by the pseudoaneurysm

**Fig. 2:**
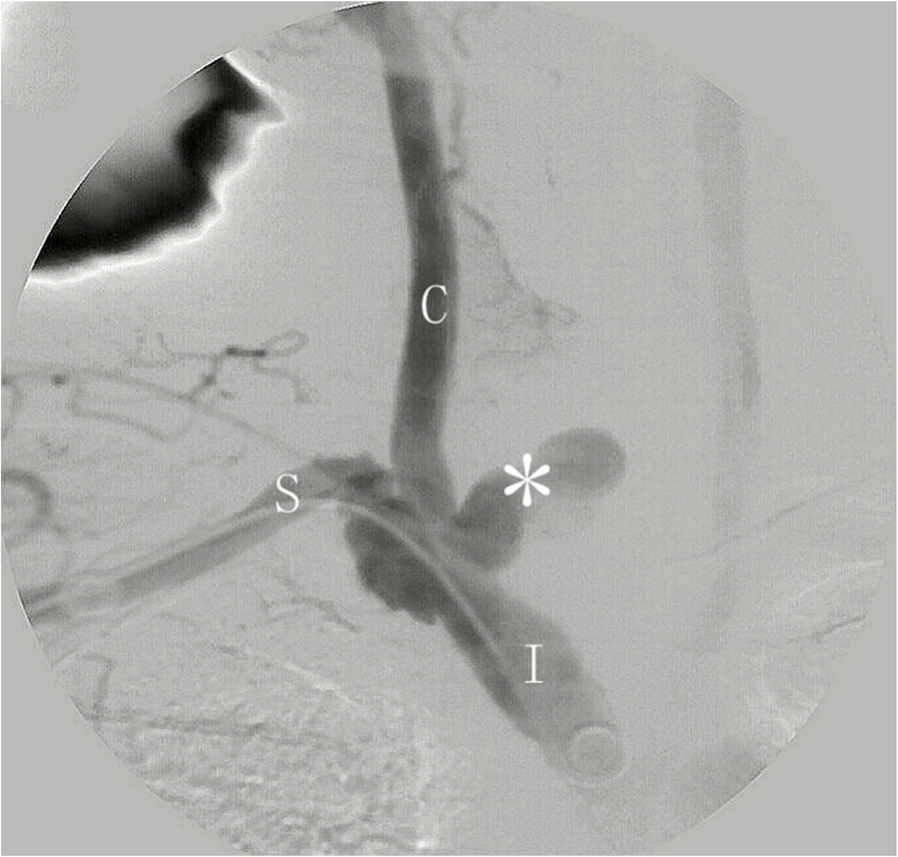
Digital subtraction angiography identifies that the rupture lesion is at the innominate bifurcation and initial of the right common carotid artery. * lesion, *C* right common carotid artery, *I* innominate artery, *S* right subclavian artery

Computed tomography angiography (CTA) showed a pseudoaneurysm at the right cervical area (Fig. 1). Contrast came from the initial location of right common carotid artery. The rupture was located on bifurcation of right common carotid artery and right subclavian artery. The diameter of the pseudoaneurysm was 4 cm. Her esophagus and trachea were slightly compressed by the pseudoaneurysm. Digital subtraction angiography (DSA) confirmed that the rupture area of the pseudoaneurysm came from innominate artery bifurcation. The artery tear lesion had a diameter of 0.5 cm (Fig. 2).

### Therapeutic intervention

Before the intervention, a monitor was used to make sure our patient’s vital signs were stable. Cardiac type B ultrasound evaluated that her cardiac function was normal. No special drug was given to her. After the preoperative preparation, the endovascular treatment was arranged in the catheter theater of our hospital. She was in a supine position. We successfully punctured her right femoral artery and right branchial artery. Then two through accesses were built using Amplatz guidewires: one was from right femoral artery to right carotid artery and the other was from right branchial artery to ascending aorta. In order to keep right carotid artery and right subclavian artery patent, two covered stent grafts from guidewire accesses were placed into innominate artery bifurcation. The two Fluency® Plus covered stent grafts (Angiomed GmbH &Co Karlsruhe, Germany) of 12 mm × 6 cm and 12 mm × 8 cm were pre-positioned in innominate artery partially, also in right carotid artery and right subclavian artery partially. A “roadmap” angiography made the delivery process precise. The two pre-positioned stent grafts were delivered and they were partially “kissing” in innominate artery. The other part of the two stent grafts was in distal site of right subclavian artery and right carotid artery separately. Balloon expansion was performed in both stent grafts. DSA after the stent grafts deployment showed the pseudoaneurysm was repaired, no contrast went into the pseudoaneurysm anymore.

### Follow-up and outcomes

**Fig. 3:**
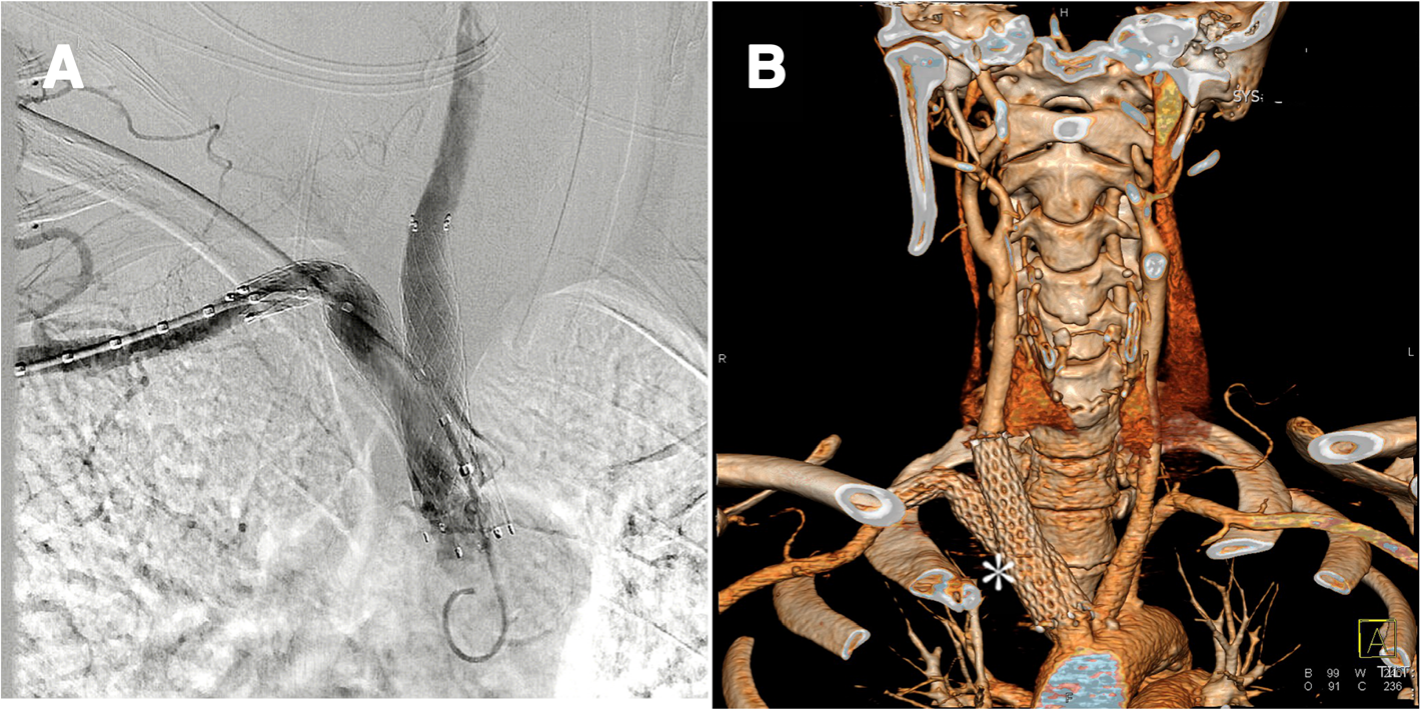
**a** After deployment of two stent grafts, digital subtraction angiography shows that no contrast goes into the pseudoaneurysm anymore, and both carotid artery and subclavian artery are patent. **b** Two-week follow-up computed tomography angiography of the patient, *white star* (*) shows the two “kissing stent grafts” are in the right position and with no contrast leak. Furthermore, both carotid artery and subclavian artery are patent

Two weeks later, CTA showed that the two stent grafts were in satisfactory position, both the right carotid artery and the right subclavian artery were patent (Fig. 3). No contrast went into the pseudoaneurysm. No pulse of the cervical mass could be palpitated after the procedure was finished. There was no need to resect the mass because the compressive symptoms had disappeared. No infection was noted at the lesion area when she came back 1 month later. Her symptoms of dysphagia and dyspnea were relieved 1–2 weeks later. She was alive and well at 1-year follow-up. She had no special complaints and she refused another CTA when we communicated with her by telephone.

## Discussion

The “kissing stent-grafts technique” we described here is migrated from kissing balloon and kissing stents in coronary artery intervention. Although the use of “kissing stent grafts” has been reported in aortic bifurcation, its target lesion is aortic-iliac artery occlusion. The use of kissing stent grafts to treat pseudoaneurysm in innominate artery bifurcation was not reported in the English and Chinese literatures that we reviewed. This is the first case report that describes endovascular repair of an innominate artery bifurcation pseudoaneurysm because the technique is not always used in bifurcation lesions other than coronary artery and aortic bifurcation. Using current devices, this technique solves the problem of large different diameter of two arteries and the isolation of the pseudoaneurysm. It is a kind of creative technique; however, deciding whether it is worth broadening its use requires more clinical data and long-term follow-up.

Pseudoaneurysm in the bifurcation of innominate artery is rarely reported. Rupture is a catastrophic result. Furthermore, massive bleeding or huge hematoma may cause lethal compression of the trachea. A lesion in this location is very difficult to deal with by open surgery. It is relatively hard to reach this deep lesion and hard to control the bleeding. In this patient, the diameter of innominate artery is 16 mm, while the diameter of right common carotid artery and right subclavian artery is 8 mm separately. Because we lack a tapered stent graft, we could not use just one stent graft to simply cover from larger diameter innominate artery to smaller diameter carotid artery even if this method would cover the right subclavian artery. The “kissing stent-grafts technique” which was inspired by kissing stents technique in coronary intervention is probably the most proper technique that can be used in this difficult situation. To our knowledge, this is the first case using kissing stent grafts to treat a pseudoaneurysm located on innominate artery bifurcation. Endovascular treatment in innominate artery is rare compared to other frequent endovascular interventions in vessels like the femoral artery or aorta. A few cases in the literature reported endovascular treatment in innominate artery and it seemed an effective and safe method [1]. Watura *et al*. reported using angioplasty to treat obstructed innominate artery early in 1995 [2]. Nagata *et al.* reported using kissing stents in innominate artery; however, they were using this procedure to treat stenosis lesion [3]. Kissing stents technique is commonly used in coronary artery intervention when it comes to bifurcation lesions. Also, an aortic-iliac occlusion lesion can be treated by this technique. Rahman and colleagues’ study showed that side branch dilation reduced the main vessel stent volume and distorted the main vessel stent symmetry in the bifurcation segment, which were restored after kissing balloon inflation [4]. They also demonstrated that kissing balloon inflation increased the main vessel stent volume and area, and induced asymmetric stent expansion in the proximal segment. In our case, because the saving of the innominate artery and the saving of the subclavian artery were of equal importance, there is no main vessel or side branch. Two covered stent grafts of the same diameter were delivered in the innominate artery side by side. We matched the transverse area of two stent grafts to innominate artery’s transverse area. It is important to use two stent grafts that correctly fit the innominate artery to avoid leakage into the pseudoaneurysm. After deploying the stent grafts, kissing balloons were applied to expand both stent grafts which can angioplasty the grafts to have a more stable fit in the bifurcation. CTA follow-up showed satisfactory early result of the isolation of the pseudoaneurysm. Our patient was relieved of her dysphagia and dyspnea symptoms and she had no cerebral symptoms, which means the instant result of this innovative treatment is acceptable. We communicated with her 1 year later by telephone. She was alive and well and refused to come back to hospital to have another CTA. However, we intend to continue follow-up and ask the patient come back to have CTA. Further follow-up results can help us to evaluate the long-term effect of this new method.

## Conclusions

Compared to conventional open surgery for an innominate bifurcation lesion, the innovative treatment using kissing stent-grafts technique is minimally invasive and has a satisfactory result. Kissing stent-grafts technique may be used in locations other than coronary and aortic bifurcation. Further follow-up results are needed to evaluate the long-term effect of this method.
